# Effect of different oxide thickness on the bending Young’s modulus of SiO_2_@SiC nanowires

**DOI:** 10.1038/srep18994

**Published:** 2016-01-07

**Authors:** Jinyao Ma, Yanping Liu, Peida Hao, Jin Wang, Yuefei Zhang

**Affiliations:** 1 College of Mechanical Engineering, Taiyuan University of Technology, Taiyuan 030024, China; 2 Institute of Microstructure and Properties of Advanced Materials, Beijing University of Technology, Beijing 100022, China

## Abstract

The surface or sheath effect on core-shell nanowires plays an important role in the nanomechanical test. In the past few years, SiC nanowires have been synthesized using various methods with an uneven and uncontrollable amorphous silicon dioxide sheath. The bending Young’s modulus of the SiC nanowires has scarcely been measured, and the effect of the oxide sheath has not been taken into account. In this paper, SiO_2_-coated SiC (SiO_2_@SiC) nanowires were synthesized using the chemical vapor deposition method, followed by thermal reduction. Scanning electron microscopy and transmission electron microscopy show that the SiO_2_@SiC nanowires in this paper have diameters ranging from 130 ~ 150 nm, with the average thickness of SiO_2_ layer approximately 14 nm. After different processing times with 1 mol/L NaOH, approximately 5 nm, 9 nm, 14 nm silicon dioxide layers were obtained. The results of the three-point-bending test show that the modulus of SiO_2_@SiC nanowires is found to clearly decrease with the increase in oxide thickness and the influence of the oxide sheath should not be ignored when the layer thickness is above 5 nm. Young’s modulus of the SiO_2_@SiC nanowires calculated in this study by the core-shell structure model is in good agreement with the theoretical value.

Over the past few years, SiC nanowires have attracted great interest due to their high mechanical strength, unique electrical and chemical properties, and other superior advantages, such as high thermal conductivity, variable band gaps, and radiation resistance. Because of their many excellent properties, SiC nanowires hold great promise for application in nano electro-mechanical systems (NEMS), cold cathode field emission displays (FEDs), nanoresonators, and semiconductor devices^1^,^2^. Although SiC nanowires are rapidly advancing, the study of the mechanical properties of SiC nanowires is still at the initial stage of development, with the potential of the materials being far from realized.

To guarantee the normal operation and reliability of SiC nanowires in relevant devices, it is very important to determine their mechanical properties, especially the Young’s modulus. Understanding the mechanical properties of SiC nanowires is of crucial importance for the successful realization of such applications and to allow us to better use and control them. Different measurement methods have been used to study the mechanical properties of SiC nanowires, including scanning electron microscopy (SEM), transmission electron microscopy (TEM)^3^,^4^, nanoindentation techniques^5^, atomistic simulations, finite element analysis and atomic force microscopy (AFM) via a three-point-bending measurement setup^6^,^7^ .

There are a considerable number of methods used to synthesize SiC nanowires, including chemical vapor deposition (CVD)^8^, physical evaporation, and laser ablation^9^,^10^. However, most of the previous studies using these methods undoubtedly reported the synthesis of SiC nanowires with uncontrollable oxide shells of different thicknesses, which is very likely to hinder the measurement of the exact value of the elastic modulus of SiC nanowires^11^. Transmission electron microscopy (TEM) imaging clearly shows that SiC nanowires synthesized via the usual methods have a core-shell structure, and energy dispersive spectroscopy (EDS) and X-ray diffraction (XRD) are used here to confirm that the core is SiC and the amorphous shell is SiO_2_, which is formed via the reaction of SiO vapor and O_2_; the amorphous SiO_2_ shell has a thickness that varies from 3 nm to 35 nm^12^,^13^,^14^. SiC nanowires with SiO_2_ layer exhibit excellent mechanical strength and super hydrophilic properties, and it will work as functional ceramic reinforcements and self-cleaning materials in the future. SiC nanowires with thin SiO_2_ layer perform a better property in field emission area^4^,^15^,^16^. However, in previous investigations of the elastic modulus, the effect of the oxide layer on the mechanical properties of silicon carbide has never been considered in details. An inspired research, Calahorra *et al.* showed that the Young’s modulus of Si nanowires is influenced by their oxide sheath, although the layer is only several nanometers thick^17^.

In this study, high-quality SiO_2_@SiC nanowires were synthesized using the CVD method followed by thermal reduction; the diameters of the nanowires ranged from 130 to 150 nm. The Young’s modulus was measured using the SEM/scanning probe microscope (SPM) combined system, and the experimental results of the three-point bending test show that the oxide layer has an obvious effect on the SiC nanowires. The experimental details expressed in the method section.

## Results and Discussion

**Figure 1:**
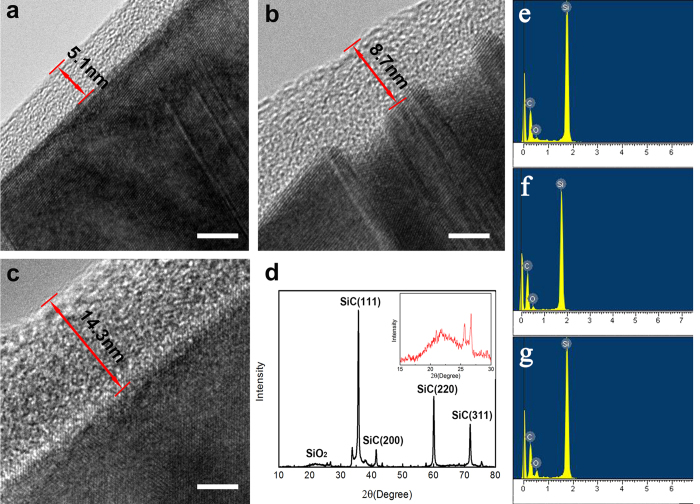
Morphology and microstructure characterization of SiO_2_@SiC nanowires. (**a–c**) TEM images of SiO_2_@SiC nanowires with different shell thicknesses. The scale bar corresponds to 5 nm. (**d**) X-ray diffraction pattern recorded from SiO_2_@SiC nanowires, and the top inset shows the enlarged image range from 15°–30°. EDS results (**e–g**) were obtained correspond to (**a–c**), respectively.

High resolution transmission electron microscopy (HRTEM) images (Fig. 1a–c) of the nanowires clearly correspond to the core-shell structure model, in which the SiC core is seemingly slightly darker than the sheath, and approximately 5 nm, 9 nm, 14 nm layers, respectively, have been obtained by different processing times with a 1 mol/L NaOH solution^11^. Next, Fig. 1(d) shows the XRD pattern of the nanowires, and the diffused XRD wave crest at low diffraction angle (15°–30°) corresponds to the amorphous-state SiO_2_ shells. The EDS results show in images (e–g) indicating the different O content which was obtained from Figure (a–c), respectively. In the original state, the thickness of the SiO_2_ oxide sheath is approximately 14 nm without any processing. To further confirm the chemical formula of the thin oxide layer, scanning transmission electron microscopy (STEM) images that were used for electron energy loss spectroscopy (EELS) line scanning were obtained using a high-angle annular dark field (HAADF) detector for TEM^18^. Finally, XRD images were obtained to confirm that the coated SiO_2_ is amorphous.

**Figure 2:**
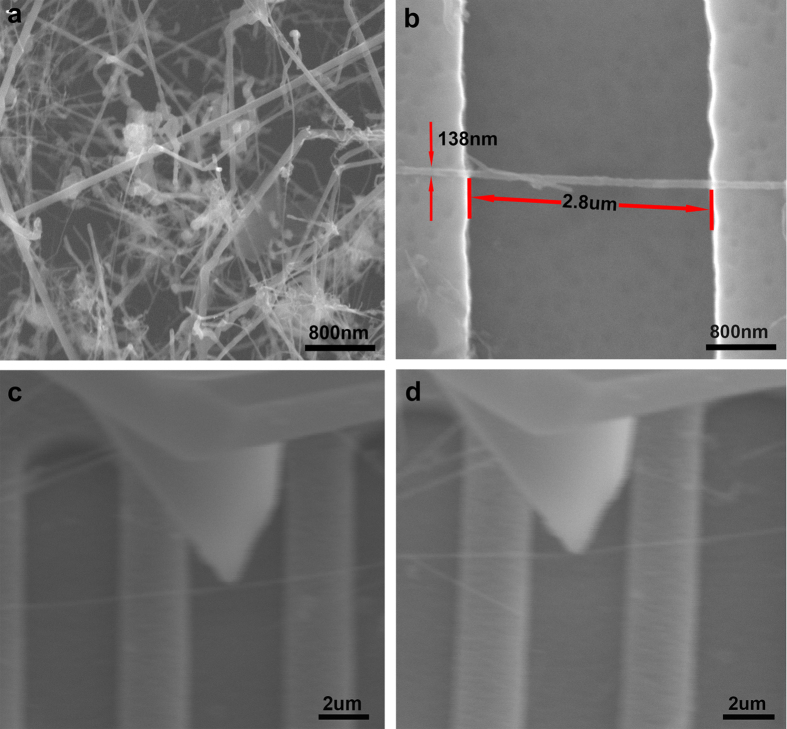
The process of three-points-bending on SiO_2_@SiC nanowires. (**a**) SEM image of the SiC nanowires morphology. (**b**) A single SiC nanowire suspended over the grooves prepared for the bending test. (**c**) SEM image of the tip almost in contact with the SiC nanowire. (**d**) The tip in contact with the SiO_2_@SiC nanowire, and the wire exhibiting bending deformation.

Figure 2(a) shows the morphology of the SiO_2_@SiC nanowires, and (b–d) correspond to the real-time image of three-point bending by an SPM tip, repeat tests were performed for different oxide thicknesses. In the image shown in (d), we can see that the nanowire has obviously been deformed. In the process, SPM tip may penetrate into the SiO2 shell. The compressive deformation leads to 10–15% error due to the tip indent into the nano spheres showed by Peida Hao *et al.*^19^, and the indentation impressions find by Xiaodong Li *et al.*^20^, In the process of indentation here, compared with their research we can see the radius of curvature at the top of the tip is much larger than SiO_2_@SiC nanowires diameters and the nanowires are overhead, there is no substrate under the nanowires. So in this paper, we ignored the depth, which the tip indents into the layer.

**Figure 3:**
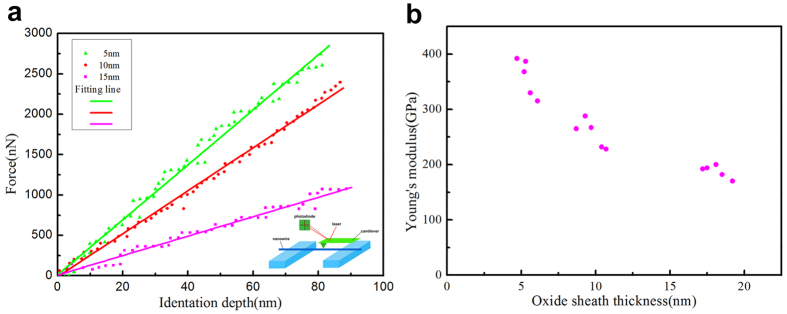
Force-displacement curves and Young’s modulus of different SiO_2_ thickness. (**a**) Fitting curves of the measured F-d plots. (**b**) Young’s modulus calculated at different sheath thicknesses.

Figure 3(a) shows three representative loading F-d curves that were obtained from the bending procedure. In the process of calculation, we neglected the influence of axial tensile stress because in our experiments, the probe force induced a small midpoint displacement; the specific relationship is given by Equation (1)^21^, i.e., the tensile stress is proportional to the cube of the midpoint deflection.


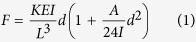


As a result, only pure bending is considered in this case. After the bending tests, the results are reproducible (Fig. 3(b)), and the fluctuations are in an acceptable range. The relationship between the Young’ modulus E and the linear elastic F-d curves are shown in Equation (2); the testing material properties are determined by assuming the Euler-Bernoulli beam model^22^:


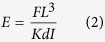


where 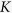 is a parameter related to boundary conditions, which is divided into two models: ends-clamped (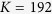) and ends-free (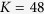). Note that if we choose the ends-free model, then the result will be four times larger than that of the ends-clamped model for the same materials. Thus, it is important to note that parameter 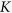 is required to make the most appropriate choice. 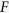 is the applied force, 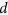 is the midpoint point displacement of the beam, 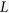 is the suspended length of SiC nanowires, the moment of inertia is 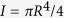 for which the section is cylindrical and 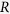 is the radius of SiC nanowires, and 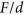 is the slope of the loading curve.

Note that for the three-point-bending test of the nanowire that is bonded to the support by adhesion, the two boundary conditions at the two ends of the nanowire can be either fixed or free. Because of the large surface-to-volume ratio, an obvious adhesion occurs; as a result, previous studies typically assume that the nanowires are fixed to their ends under experimental conditions^23^,^24^,^25^,^26^. However, there is no theoretical basis for the hypothesis of these tests, and inappropriate boundary conditions can cause systematic errors for the results of the bending modulus. The formulas described here are related to many factors, especially the softening/adhesive effect, the applied force magnitude and the nanowire geometry.

The aforementioned factors that influence the parameter K require further analysis. Figure 2 shows that in our model, SiO_2_@SiC nanowires are suspended over a trench without being fixed at the ends, the diameters of the wires range from 130–150 nm, the widths of the trenches are approximately 2.6–3.2 μm, and the core-shell nanowires are relatively harder. Considering the specific conditions of this experiment and in reference to previous studies^27^, 48 has been selected here for parameter 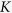. Later in this study, we will further verify the selection of 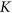.

The Young’s modulus calculated above is not that of the pure SiC nanowires because it is actually a composite modulus that comprises both the SiC core and the SiO_2_ shell. According to the core-shell theoretical model^17^, the relationship between the composite modulus and bending test’s modulus is given by Equation (3):


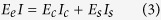


where 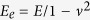 is the effective Young’ modulus, 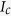 is the moment of inertia about the SiC core, 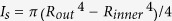 is the cylindrical shell of SiO_2_, and 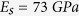 is the shell of SiO_2_^27^ Here, we consider 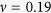 as the Poisson’s ratio of the pure SiC nanowires. 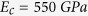 in this study is considered for the bulk SiC because the Young’s modulus of SiC nanowires is 603 GPa, as obtained by Lambrecht *et al.*^28^, while it is 558 GPa, as calculated by Li *et al.*^29^, and it is 581 GPa with 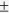
10% scatter, as measured by Petrovic *et al.*^30^. The results above are identical to modulus of bulk SiC (503–600 GPa) and are basically not influenced by size^14^; thus, we regard it as a known parameter of the average modulus of bulk SiC.

**Figure 4:**
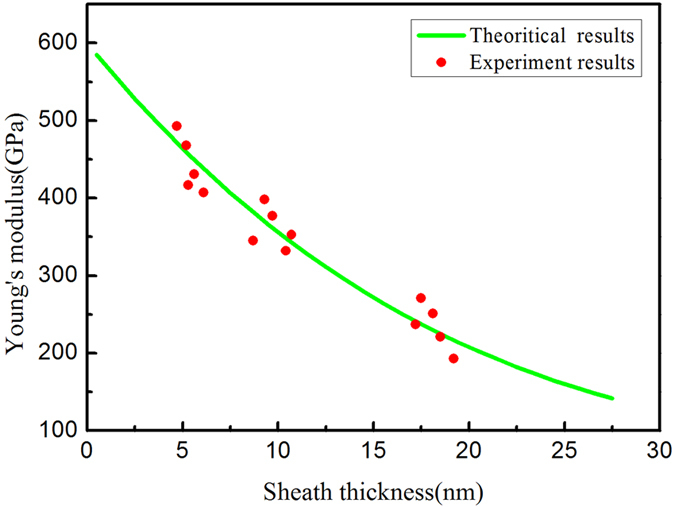
Continuous curve for the oxide thickness and the Young’s modulus of the core-shell SiC nanowires.

Through a certain transformation, the formula can be simplified, as shown in Equation (4):


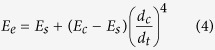


where 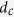 is the SiC core diameter, and 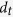 is the total diameter. From Equation (3), we find that even a very thin shell will have a great effect on 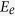. Using Equation (3), we can deduce the value of 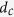, and the thickness of oxide sheath can be calculated using Equation (5):


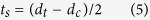


where 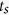 is the thickness of the SiO_2_ oxide sheath. From Equations (3), (4), and (5) above, the relationship between 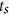 and the Young’s modulus of SiO_2_@SiC nanowires is shown in Fig. 3(b). The result of 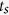 agrees well with the observed TEM image regarding the oxide sheath thickness. If we take the average value of 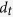 to be a constant parameter, then a continuous curve for the oxide thickness and the Young’s modulus of the core-shell SiC nanowires can be obtained in Fig. 4, and the fitting curve is in good agreement with the modulus obtained from the test. At the same time, this curve not only makes us more aware of the trend of this change but also verifies that the parameter 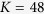 used from the beginning of this study is suitable.

Equipped with the theoretical model and the fitting procedure, Table 1 presents the average value of the composite Young’s modulus of the core-shell SiC nanowires and the oxide layer thickness.

The bending Young’s modulus values of 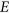 for SiO_2_@SiC structured nanowires with different oxide layers are rather different from those reported in Table 1. The experimental data are nicely scattered along with the theoretical value and the oxide sheath observed in the TEM images. In actually, SiC nanowires or film in experiment usually have a certain thickness of oxide layer and this is why the value of bulk or film SiC obtained before smaller than the theoretical value. The bending modulus for SiC nanowires with 5 nm thickness in ou paper agrees well with the bulk and film SiC measured in previous research^31^,^32^, and this can more approve the correctness of our results.

These results clearly show that when the thickness of the SiO_2_ layer increases, the value of 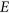 decreases.

## Conclusions

In previous experiments, the value of the bending Young’s modulus of SiC nanowires had no consensus and was considered to be influenced by the microstructures of SiC nanowires. However, these investigations typically ignored the effect of the oxide layer in the calculation of the Young’s modulus. In this study, the images taken by TEM, EDS and XRD confirmed that SiC nanowires synthesized using the CVD method have a SiO_2_ oxide sheath. Using different processing times in NaOH solution, we obtained SiO_2_@SiC nanowires with different thicknesses of the oxide sheath. Further more, we investigated the modulus of core-shell structured SiO_2_@SiC nanowires via the three-point-bending method. *In situ* quantitative test results obtained using the SEM/SPM combined system show that when the core-shell SiC nanowire’s oxide coating thickness changes by approximately 5 nm, the value of the Young’s modulus will produce 16% changes on average. Thus, the oxide layer has a certain effect on the modulus of SiC nanowires, despite the thickness of the sheath being very thin.

## Methods

### Materials

The high-quality SiC nanowires in this study were synthesized using the CVD method followed by thermal reduction. SiC nanowires with three different oxide thicknesses were determined by using different processing times with a 1 mol/L NaOH solution^11^.

### Characterizations

TEM images and EDS were obtained using a JEOL-2010 F instrument; SEM images were obtained using a Quanta 250 instrument made by FEI company. XRD data were obtained using a D8 ADVANCE instrument produced in Germany. Here, we employed a SEM/SPM combined testing system like Hao, P. *et al.* describe in their paper^19^, and using the three-point-bending method to measure the Young’s modulus of the SiO_2_@SiC nanowires.

### Three-point-bending mechanical test

Core-shell nanowires with three different oxide layer thicknesses were dropped onto clean Si substrates with regular trenches; the trenches were fabricated using photolithography and reactive ion etching. The measurements were performed on suspended SiO_2_@SiC nanowires subjected to home-made SEM/SPM *in-situ* three-point-bending constraints. By real-time SEM image, it is preferable to choose nanowires oriented perpendicular to the bending. The nanowires position was adjusted in three different directions via piezoelectric ceramic actuators until SPM tip was achieved with the middle point of the respective nanowires. And then, approaching the nanowire to the SPM tip performs the *in-situ* bending experiment. When the SPM tip contacting the nanowire, there was a change in laser path as well as the signal of cantilever deflection. A more detailed description of the experimental setup previously can be found in the recent literature^14^,^19^. However, note that we did not fix the nanowires to both ends of the trench. During the process, we can manipulate the relative position between the tip and SiC nanowires using SPM and we can also observe the real-time SEM image. Here, we repeated the bending process several times for SiC nanowires with the same oxide thickness to ensure that the results are reliable.

## Additional Information

**How to cite this article**: Ma, J. *et al.* Effect of different oxide thickness on the bending Young’s modulus of SiO_2_@SiC nanowires. *Sci. Rep.* **6**, 18994; doi: 10.1038/srep18994 (2016).

## Figures and Tables

**Table 1:** The average bending Young’s modulus of core-shell SiC nanowires calculated using the three-point-bending model for the SEM/SPM combined system and with the oxide sheath thickness extracted using Equations (3) and (4).

Bending Young’s modulus 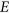 (GPa)	443.2	361.3	234.6
Oxide sheath thickness 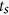 (nm)	5.33	8.75	17.52
